# *Aucuba japonica* extract inhibits retinal neovascularization in a mouse model of oxygen‐induced retinopathy, with its bioactive components preventing VEGF‐induced retinal vascular hyperpermeability

**DOI:** 10.1002/fsn3.1590

**Published:** 2020-04-30

**Authors:** Eunsoo Jung, Woo Kwon Jung, Su‐Bin Park, Hyung Rae Kim, Junghyun Kim

**Affiliations:** ^1^ Laboratory of Toxicology Research Institute for Veterinary Science and College of Veterinary Medicine Seoul National University Seoul Korea; ^2^ Department of Oral Pathology School of Dentistry Jeonbuk National University Jeonju Korea

**Keywords:** angiogenesis, *Aucuba japonica*, functional food, retinal neovascularization

## Abstract

Neovascularization in the retina is common pathophysiology of diabetic retinal microvasculopathy and exudative macular degeneration. Our study assessed the inhibitory activity of an ethanol‐based extract of *Aucuba japonica* (AJE) on abnormal angiogenesis in the retina with a hyperoxia‐induced neovascular retinopathy model. The inhibitory effects of aucubin, quercetin, and kaempferol, bioactive compounds, from *A. japonica*, on retinal vascular hyperpermeability were also examined. On the 7th postnatal day (P7), the C57BL/6 pups were exposed to a hyperoxic environment with 75% oxygen to develop the experimental angiogenesis in retinas. On the 12th postnatal day (P12), the pups were then returned to the normal atmospheric pressure of oxygen. From P12 to P16, the administration was intraperitoneal. The dose per day was 250 mg per kg weight. Retinal neovascularization was measured with retinal flat mounts prepared on P17. We also measured the vascular leakage mediated by the vascular endothelial growth factor (VEGF) in retinas. Mice treated with AJE had markedly smaller neovascular lesions, in comparison with vehicle‐administered mice. AJE downregulated the expression of both VEGF protein and mRNA. In addition, aucubin, quercetin, and kaempferol ameliorated VEGF‐induced retinal vascular leakage. The results of our study suggest that AJE is a potent antiangiogenic substance. AJE could also serve as a therapeutic agent for abnormal growth of vessels in the retina in patients with ischemic retinopathy. The bioactive compounds of AJE may be responsible for its antiangiogenic abilities.

## Graphical abstract

*Aucuba japonica* extract (AJE) and its bioactive compounds, aucubin, quercetin, and kaempferol, showed potent anti‐angiogenic activities in a mouse model for oxygen‐induced retinopathy. This study proposes AJE as an anti‐angiogenic functional food for patients with abnormal retinal vessel growth.

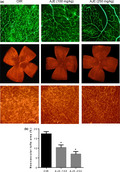

## 1 INTRODUCTION

Advanced retinal neovascularization causes visual impairment and a complete loss of sight in a significant proportion of the elderly over 65 years of age (Solomon, Lindsley, Vedula, Krzystolik, & Hawkins, 2019). Retinal neovascularization is also implicated in severe complications of retrolental fibroplasia, diabetic retinal microvasculopathy, and neovascular macular degeneration (Campochiaro, 2013).

A prominent proangiogenic and vascular permeability factor is the vascular endothelial growth factor (VEGF). VEGF also plays a crucial mediating role in the pathogenesis of these retinal diseases (Parikh et al., 2019). The use of anti‐VEGF agents to inhibit the VEGF signaling pathway has recently successfully reduced retinal neovascularization in human subjects (Eyetech Study Group, 2003) and animal models (Muranaka et al., 2005).

Anti‐VEGF drugs, such as bevacizumab, aflibercept, and ranibizumab, have been administered intravitreally in clinical trials. The drugs caused significant neovascularization suppression and vision loss stability (Campa & Harding, 2011; Frampton, 2013; Garcia‐Layana et al., 2015). It is noteworthy that the intravitreal administration of anti‐VEGF agents has been associated with adverse effects (Diago et al., 2009; Fintak et al., 2008). When anti‐VEGF agents are administered intravitreally repeatedly, ocular complications, such as endophthalmitis, traumatic cataracts, ocular inflammation, retinal detachment, intraocular pressure elevation, and vitreous hemorrhage, can occur at a high incidence (Falavarjani & Nguyen, 2013). Novel agents using other administration routes are thus increasingly considered (Cammalleri et al., 2017; Honda et al., 2010; Meredith et al., 2015; Takahashi et al., 2008).

Some food supplements and its ingredients have been considered to be inhibitors of ocular angiogenesis (Sulaiman, Basavarajappa, & Corson, 2014) and retinal degeneration (Dal Monte et al., 2018; Locri, Cammalleri, Dal Monte, Rusciano, & Bagnoli, 2019). *Aucuba japonica* Thunb. has been used as a functional food to treat several diseases, such as edema and inflammation in Korea and Japan (Kimura, But, Guo, & Sung, 1997). The leaves of *A. japonica* have various phytochemicals such as aucubin, quercetin, and kaempferol (Bernini, Iavarone, & Trogolo, 1984; Iwashina, Kamenosono, & Hatta, 1997). Recently, we reported that *A. japonica* and its bioactive compound, aucubin, showed potent pharmacological effects on dry eye disease (Kang, Jung, & Kim, 2018).

The antiangiogenic abilities of *A. japonica* on the neovascular retinal diseases have not been described in reports, according to our research. To elucidate this, we examined the antiangiogenic activities of an ethanolic extract of *A. japonica* (AJE) in an oxygen‐induced ischemic retinopathy (OIR) model. The ability of aucubin, kaempferol, and quercetin to inhibit retinal vascular hyperpermeability stimulated by administering exogenous VEGF intravitreally in rats was also assessed.

## 2 MATERIALS AND METHODS

### 2.1 AJE preparation

**FIGURE 1:**
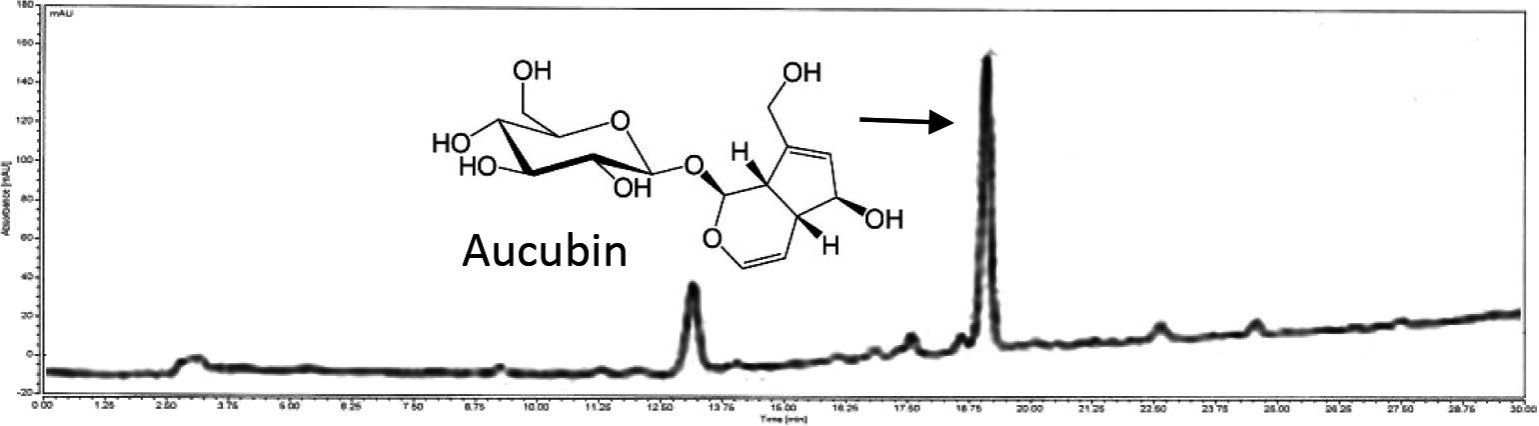
HPLC profile of an extract of *Aucuba japonica*

The leaves and stems of *A. japonica* were cultivated and collected in Geoje, Kyungsangnamdo, South Korea. Jeonbuk National University's (Jeonju, South Korea) herbarium has the voucher specimen (No. JBNU‐AJE2018). The leaves (700 g) and stems (350 g) of *A. japonica* were extracted with 30% ethanol (10.5 L) at 85°C. The extraction took 3 hr with 175 g sample gotten by concentration and freeze‐drying. AJE was qualitatively and quantitatively assessed with high‐performance liquid chromatography (HPLC). AJE contained 59.7 ± 1.5 mg/g aucubin (Figure 1).

### 2.2 OIR model experimental neovascularization of the retina

As earlier described, the study was carried out C57BL/6 pups, with neovascularization of the retina stimulated in them (Lee et al., 2013). While with their nursing mothers, on the 7th postnatal day, the pups were exposed to 75% oxygen. The exposure lasted 5 days, and they were returned to normal oxygen exposure on P12. Four groups were then created, and the pups randomly allocated to these groups, with seven mice in each group. The groups are (a) OIR model mice; (b) OIR model mice administered 100 mg of AJE per kg body weight; and (c) OIR model mice administered 250 mg AJE per body weight. The intraperitoneal administration of AJE was carried out from P12 to P16. An equal volume of the vehicle was also administered to the mice for 5 days. The Institutional Animal Care and Use Committee approved the use of the animals as well as the care (Approval No. 19‐015).

### 2.3 Neovascular area analysis with isolectin staining and fluorescein–dextran angiography

The inhalation of isoflurane anesthetized the mice. This was carried out on the 17th postnatal day. The heart of the mice was also injected with 10 mg per body weight of fluorescein–dextran (FD40, Sigma). For 1.5 hr, the eyeballs were immersed in 4% paraformaldehyde after they had been enucleated. This process occurred 5 min after the cardiac injection. Mounts of whole retinas were made on microscopic slides after isolation. With a fluorescence microscope (BX51, Olympus), the mounts were viewed. With the ImageJ program (National Institutes of Health), we measured the part of the retina that had been vas‐obliterated. The stain for the neurovascular area of the retina was rhodamine‐conjugated isolectin B4 (Vector Laboratories Ltd.). Lecithin‐based areas were identified with a fluorescence microscope. The ImageJ program was also applied in calculating the size of the neurovascular area.

### 2.4 Real‐time PCR

The weight of frozen samples of the retina was determined. TRIzol solution (Invitrogen Inc.) was applied for isolating the total RNA. An existing protocol was applied in the real‐time PCR (Lee et al., 2016). Table 1 shows the primers for GAPDH and VEGF. The Bio‐Rad iQ5 software (Bio‐Rad Laboratories Inc.) was applied in determining the mRNA levels of VEGF.

**Table 1:** Primer sequences for real‐time PCR analysis

Genes	Primers	Sequences
VEGF	Forward	5′‐TCCTCCTATCTCCACCACCTATCC‐3′
	Reverse	5′‐GACCCAGCCAGCCATACCC‐3′
GAPDH	Forward	5′‐AACGACCCCTTCATTGAC‐3′
	Reverse	5′‐TCCACGACATACTCAGCAC‐3′

### 2.5 Immunohistochemical staining

At necropsy, with the eyes fixed and embedded in 4% paraformaldehyde and paraffin respectively, retina sections 4 μm thick were made. The sections were then deparaffinized, rehydrated, and treated. Xylene deparaffinized the subjects, and 1% H_2_O_2_ in methanol was used for treating them. Incubation with anti‐VEGF antibody (Catalog No. ab1316, Abcam) which lasted for 1 hr at 37°C followed. An Envision kit (DAKO) detected the signal which was visualized with 3,3′‐diaminobenzidine tetrahydrochloride.

### 2.6 Vascular hyperpermeability of retina induced by VEGF

The induction followed a published protocol (Lara‐Castillo et al., 2009). *SD* rats that are 7 weeks old were bought from Koatech and anesthetized using isoflurane. A total of 4 μl of a single dose of 100 ng VEGF164 (R&D Systems) was administered into the vitreous cavity of one eye with a microinjector (Hamilton). The other eye was injected with the same volume of physiological saline. The following five groups of rats were then created as follows: (a) rats injected intravitreally; (b) rats injected intravitreally and treated with 100 mg of AJE per kg body weight; (c) rats injected intravitreally and exposed to 100 mg of aucubin per body weight; (d) rats injected intravitreally and treated with 100 mg per kg body weight of quercetin; and (e) rats injected intravitreally and treated with 100 mg per kg body weight of kaempferol. After the rats were injected intraocularly, AJE, quercetin, aucubin, and kaempferol were administered once every day for 3 days. The quantification of the extravasated tracer dye was done using a method described previously (Jung, Kim, Kim, Kim, & Cho, 2015).

### 2.7 Statistical analysis

One‐way analysis of variance then Tukey's multiple comparison test was applied for group data analysis. A statistically significant difference was indicated by a *p*‐value <.05.

## 3 RESULTS AND DISCUSSION

### 3.1 AJE inhibits OIR model retinal neovascularization

**FIGURE 2:**
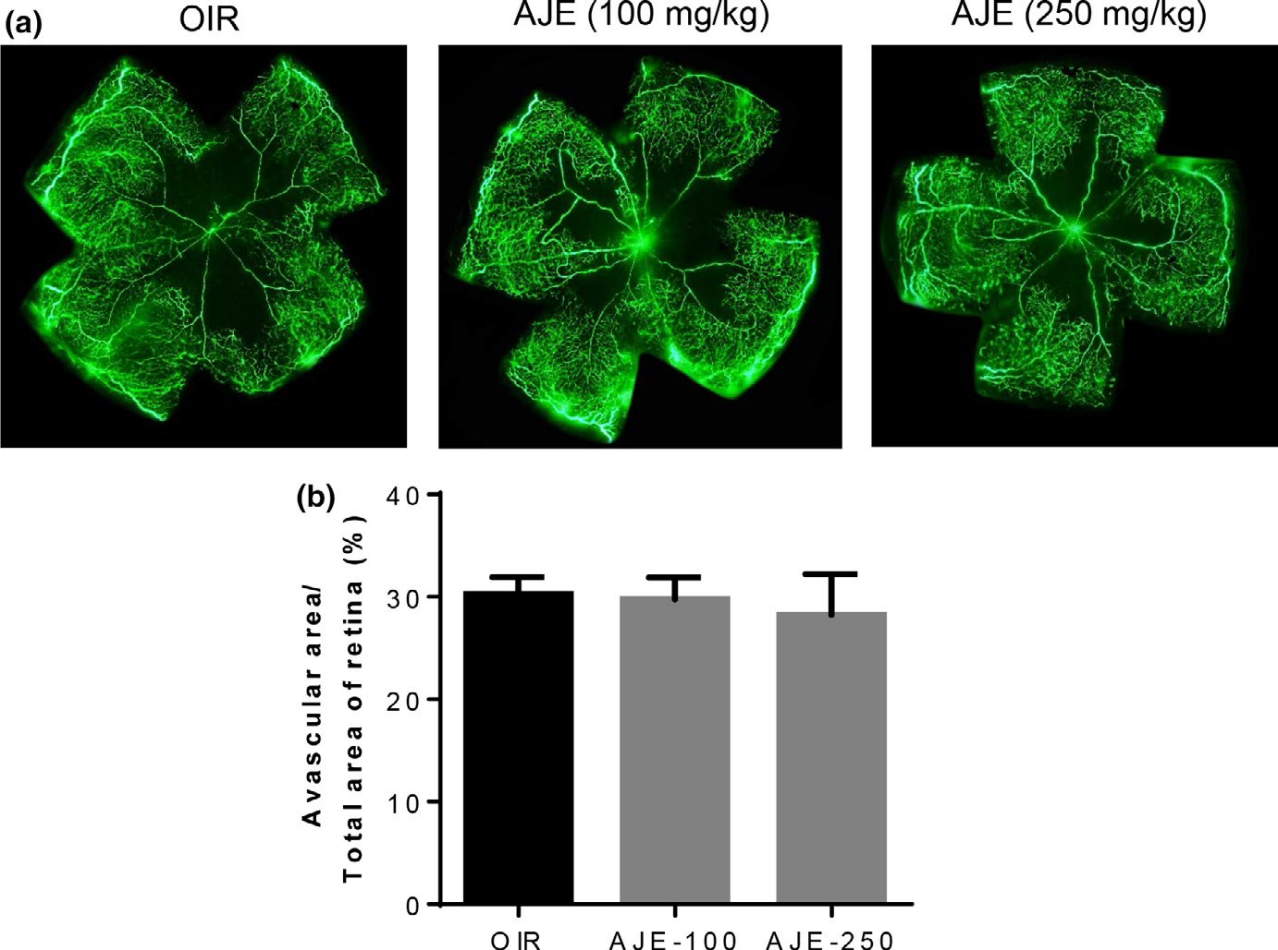
The effect of AJE on vascular obliteration of the central retina in OIR mice. (a) The retinal blood vessels were visualized via fluorescein angiography using FITC–dextran. (b) The quantification results are expressed as the percentage of the central nonperfused area within the total retinal area. The bar graph values represent the mean ± *SEM*, *n* = 7

Retinal degenerative diseases such as wet form macular degeneration and diabetic retinopathy result in severe vision loss because of abnormal angiogenesis (Gehrs, Anderson, Johnson, & Hageman, 2006). VEGF signaling pathways have been implicated in the pathogenesis of several retinal diseases (Aiello, 1997). There is thus major interest in disrupting VEGF signaling in producing new drug candidates (van Wijngaarden & Qureshi, 2008). This study focused on demonstrating how AJE influences neovascularization, using OIR model. Suppression of VEGF expression occurred in OIR model in the hyperoxic phase (P7‐P12) (Stone et al., 1996). The upregulatory influence on VEGF was observed under normal conditions (Ashton, 1957, 1966). For ischemic retinopathy (P12‐P17), the significant upregulation of proangiogenic VEGF mRNA translation triggered the development of abnormal neovascularization (Hoeben et al., 2004). VEGF has been well‐recognized as a major regulator of vascular changes due to pathology, and its inhibition halts these changes (Aiello et al., 1994; Dorey, Aouididi, Reynaud, Dvorak, & Brown, 1996). As shown in Figure 2, the vascular loss occurred in mice where hyperoxic condition (P7‐P12) was induced with nonperfused areas. The ischemic condition (P12‐P17) triggers the development of abnormal neovascularization. Neovessels are characterized by increased permeability caused by underdeveloped interendothelial junctions and incomplete basement membrane. In the present study, the fluorescein–dextran was perfused for 5 min in the OIR mice. For this reason, we detected a small amount of vascular leakage from neovessels in the fluorescein–dextran angiography.

**FIGURE 3:**
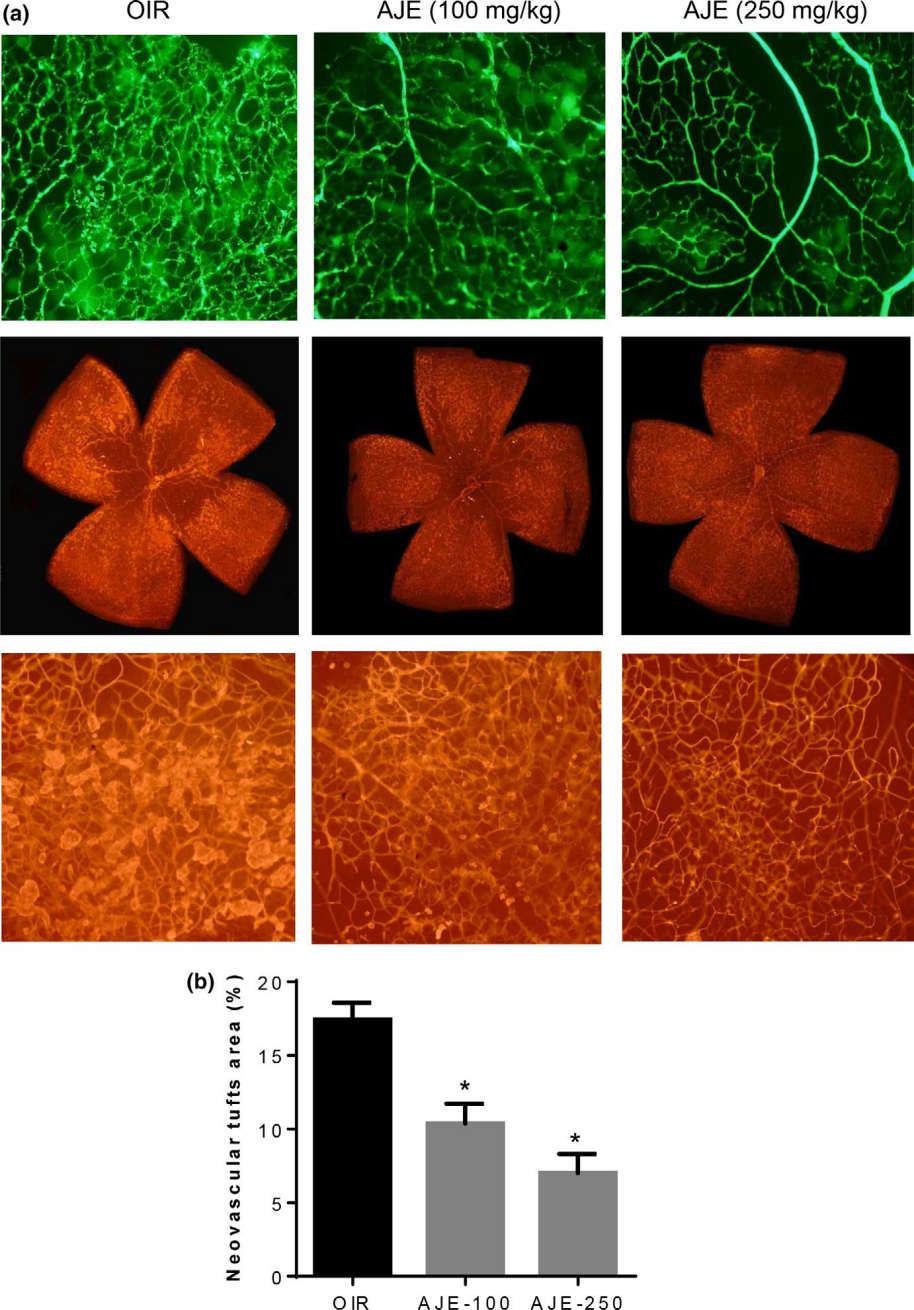
The effect of AJE on retinal neovascularization in OIR mice. (a) The retinal neovascular tufts were visualized using isolectin B4 staining. (b) Quantification results are expressed as neovascular tufts on the retina surface. The bar graph values represent the mean ± *SEM*, *n* = 7, *p < .05 versus OIR mice

Stained with isolectin B4, the neovascular areas formed were identified with immunofluorescence. OIR mice treated with AJE had fewer retinal vascular changes. Figure 2 showed that exposure to AJE failed to induce significant changes in vascular loss. At 100 and 250 mg per kg weight exposure daily, AJE stopped the development of neovascular areas by 40.3 ± 2.5% and 59.8 ± 2.9% (Figure 3). According to these findings, AJE treatment significantly reduces the size of neovascular tufts.

Although AJE has been usually administered orally in humans, we used intraperitoneal injection as the route of administration. The oral route is the most convenient route of administration. However, the neonatal mouse has historically used the intraperitoneal route of administration for dosing (McClain et al., 2001). There is no method of minimally invasive drug delivery that can accurately and reliably deliver drug compounds orally to neonatal mice (Butchbach, Edwards, Schussler, & Burghes, 2007).

### 3.2 AJE downregulates VEGF expression

**FIGURE 4:**
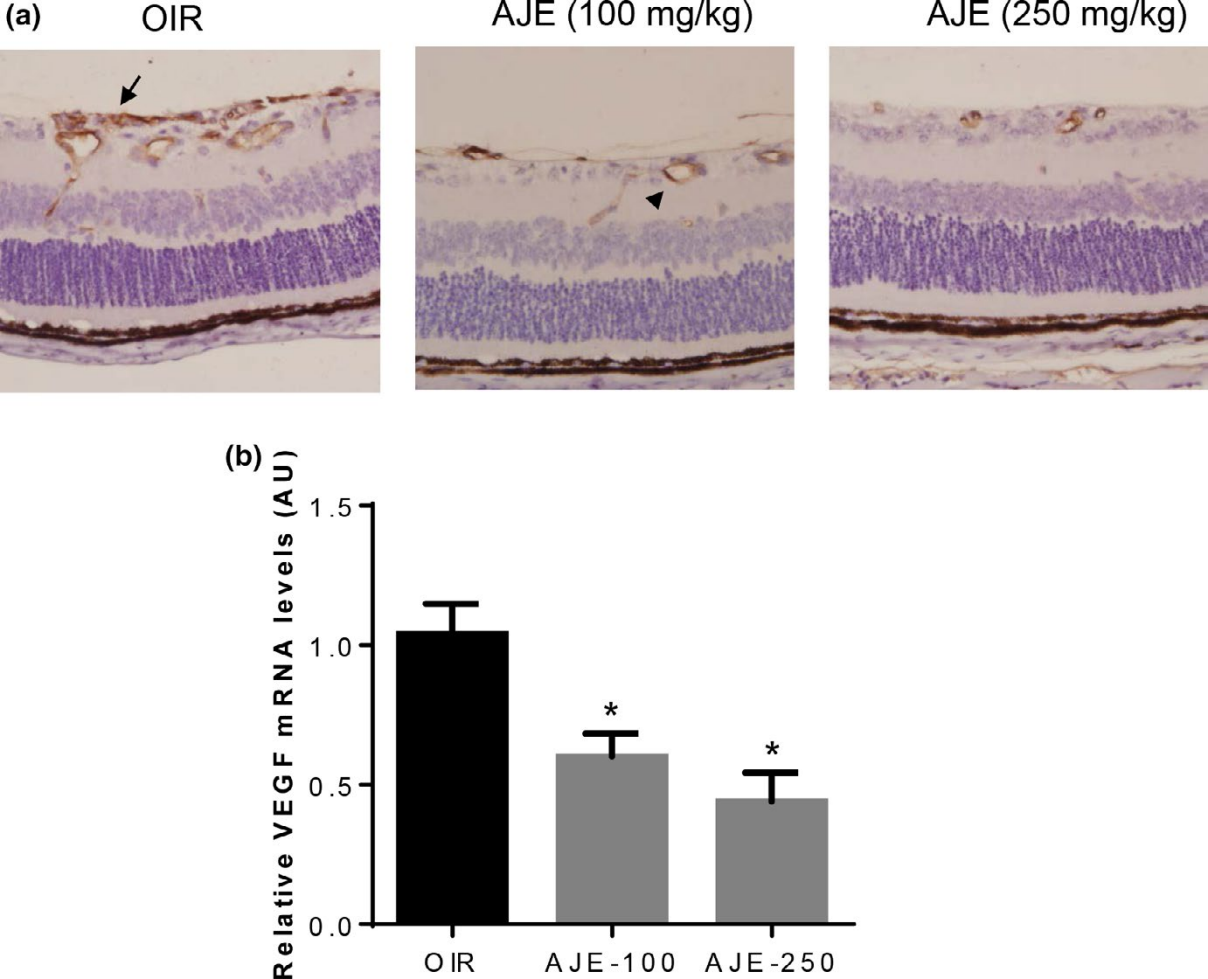
The effect of AJE on VEGF expression in OIR mice. (a) Immunohistochemical staining for VEGF in the retinal section. Arrow indicates endothelial cells of neovessels on the surface of the retina stain intensely for VEGF. Arrowhead indicates blood vessels positively stained by VEGF within the retina. (b) Real‐time PCR analysis of VEGF mRNA levels in OIR mice. The data are shown as the mean ± *SEM*, *n* = 7, *p < .05 versus OIR mice

Vascular endothelial growth factor is well known for vascular permeability. VEGF is also known as a proangiogenic molecule for inducing proliferation of the endothelium, migration, and angiogenesis (Shweiki, Itin, Soffer, & Keshet, 1992). Several VEGF‐inhibiting drugs have benefited patients with neovascular macular degeneration and proliferative diabetic retinopathy (Campochiaro, 2013; Dhoot & Avery, 2016). Real‐time PCR and immunohistochemistry were applied in assessing the difference in VEGF expression in the retina. Figure 4a shows that the immunohistochemical staining of retinal sections revealed endothelial cells of neovessels on the surface of the retina staining intensely for VEGF (arrows) that may be contributing to the neovascularization. In the AJE‐treated groups, VEGF‐positive signals were mostly detected in blood vessels within the retina (arrowheads). As predicted, the VEGF mRNA levels were markedly decreased by the treatment of AJE during ischemic retinopathy compared to that of the OIR group (Figure 4b).

Regarding cellular mechanisms for suppressing retinal neovascularization by the treatment with AJE, the present data showed that the treatment led to significant suppression of VEGF expression. In previous reports, macrophages, a rich source of VEGF, have been shown to facilitate the development of retinal neovascularization (Kvanta, Algvere, Berglin, & Seregard, 1996). Macrophages contribute to and participate in inflammatory processes that exacerbate new vessel formation. It has been reported that AJE has anti‐inflammatory activity through inhibiting the overexpression of IL‐1β, IL‐8, and TNF‐α in corneal epithelial cells (Kang et al., 2018). Collectively, the currently observed suppression of retinal neovascularization by the treatment with AJE is likely attributable to the inhibition of VEGF secretion and its anti‐inflammatory activity.

### 3.3 AJE and its bioactive compounds inhibit vascular hyperpermeability induced by VEGF

**FIGURE 5:**
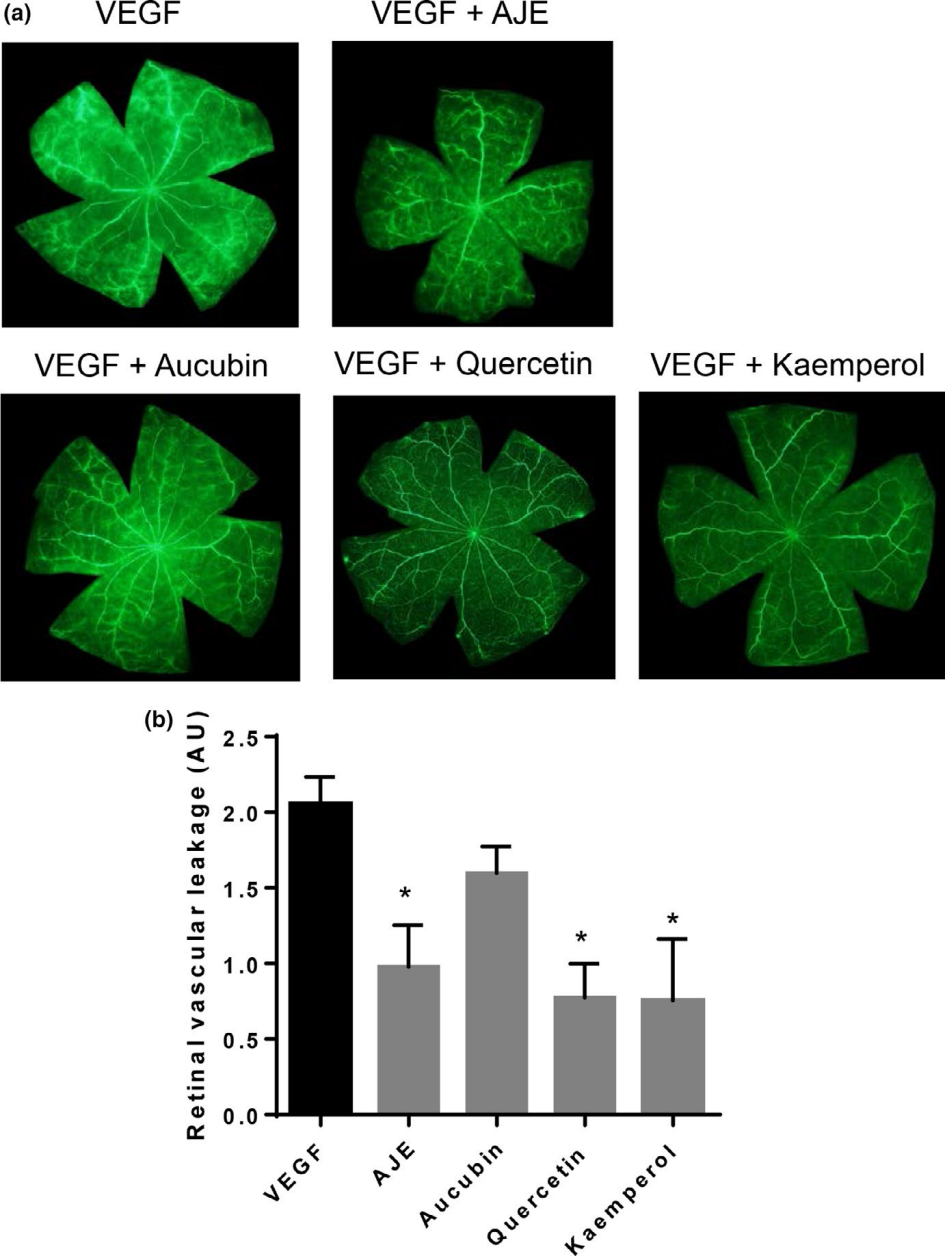
The effect of AJE and its bioactive compounds on VEGF‐induced retinal vascular leakage. (a) FITC–dextran angiography on retinal flat mounts. (b) Quantitative analysis of retinal vascular permeability. Values in the bar graphs represent the mean ± SE, *n* = 5. *p < .05 versus VEGF

Aucubin is a major compound of AJE. Owashina et al. reported that quercetin and kaempferol are bioactive flavones from *A. japonica* (Iwashina et al., 1997). To determine how AJE influences the induction of vascular pathological change by VEGF in retinas, fluorescein angiography was carried out in exogenous VEGF, with the rats injected intravitreally. The control samples retained fluorescence dye in the vessels. When exogenous VEGF was administered intravitreally, it caused leakage in several areas. To test whether AJE interferes with VEGF signaling in the retinas resulting in suppression of retinal vascular hyperpermeability, rats were administered with AJE and its bioactive compounds for 3 days after intravitreal injection of VEGF. AJE, quercetin, and kaempferol markedly reduced the retinal vascular leakage of fluorescein–dextran (Figure 5). Aucubin exhibited a minimal reduction of vascular permeability in the retina of eyes injected with VEGF. AJE and its bioactive compounds, aucubin, quercetin, and kaempferol, also inhibit VEGF‐mediated retinal vascular leakage in rats. Quercetin suppressed excessive inflammation induced by VEGF in retinal photoreceptor cells (Lee, Yun, Lee, & Yang, 2017). The formation of tubes of rhesus macaque choroid‐retinal endothelial cells was also inhibited by quercetin (Li et al., 2015). Quercetin also has antiangiogenic activities by targeting VEGF in retinopathy of prematurity induced in rodents and human retinoblastoma cells (Song, Zhao, Xu, & Zhang, 2017). Kaempferol suppressed angiogenetic activities of human retinal endothelial cells via targeting VEGF during high‐glucose condition (Xu, Zhao, Peng, Xie, & Liu, 2017) and inhibited the upregulation of VEGF mRNA stimulated by hydrogen peroxide in retinal pigment epithelial cells of humans (Du, An, He, Zhang, & He, 2018). Aucubin is a major compound of AJE and has various pharmacological activities, including antiproliferation, by blocking cell cycle progression (Hung, Yang, Tsai, Huang, & Huang, 2008) and gastroprotection by normalizing the level of VEGF (Yang et al., 2017). Although details on how AJE works are still limited, there are indications that angio‐supressive activity of AJE may be due to the kaempferol and quercetin components.

In the OIR mouse model, AJE reduced VEGF mRNA and protein levels. This result suggests that AJE has an inhibitory potency on the upstream signaling pathway of VEGF. However, in the intravitreal VEGF‐injected rat model, this result suggests that AJE and its components also have potent inhibitory activities on the downstream signaling pathway of VEGF. Although concrete information is limited, AJE may have inhibitory activities on both the upstream and downstream signaling pathways of VEGF.

In traditional herbal medicines, herbal extracts were known to have various advantages of synergy and interactions among the various phytocompounds present in the herbs. Therefore, the focus of this study is to elucidate the molecular mechanism of AJE, not each single compound. Although the actual amount of active compound had no inhibitory activity against VEGF‐induced vascular hyperpermeability, angio‐supressive activity of AJE may be due to the synergistic effect among kaempferol and quercetin.

## 4 CONCLUSION

To the best of our knowledge, our work is the first to show that AJE significantly suppressed exudative and neovascular retinopathy in vivo. The effect of AJE on the translation of VEGF mRNA was also demonstrated using a mouse model of experimental OIR. In addition, AJE and its bioactive compounds, quercetin and kaempferol, also inhibit VEGF‐mediated retinal vascular leakage in rats. Cumulatively, our findings indicate that the angio‐suppressive activities of AJE in the retina are as a result of the potency of bioactive compounds quercetin and kaempferol. Therefore, this study proposes AJE as an antiangiogenic functional food for patients with abnormal retinal vessel growth.

## CONFLICT OF INTEREST

The author declares that I do not have any conflict of interest.

## ETHICAL APPROVAL

All applicable institutional guidelines for the care and use of animals were followed.
